# Femtosecond diffraction studies of solid and liquid phase changes in shock-compressed bismuth

**DOI:** 10.1038/s41598-018-35260-3

**Published:** 2018-11-16

**Authors:** M. G. Gorman, A. L. Coleman, R. Briggs, R. S. McWilliams, D. McGonegle, C. A. Bolme, A. E. Gleason, E. Galtier, H. J. Lee, E. Granados, M. Śliwa, C. Sanloup, S. Rothman, D. E. Fratanduono, R. F. Smith, G. W. Collins, J. H. Eggert, J. S. Wark, M. I. McMahon

**Affiliations:** 1 SUPA, School of Physics & Astronomy, and Centre for Science at Extreme Conditions, The University of Edinburgh, Edinburgh, EH9 3FD UK; 2 Lawrence Livermore National Laboratory, 7000 East Avenue, Livermore, CA 94500 USA; 3 Department of Physics, Clarendon Laboratory, University of Oxford, Parks Road, Oxford, OX1 3PU UK; 4 Shock and Detonation Physics, Los Alamos National Laboratory, P.O. Box 1663, Los Alamos, New Mexico 87545 USA; 5 Stanford Institute for Materials and Energy Sciences, SLAC National Accelerator Laboratory, Menlo Park, California 94025 USA; 6 Linac Coherent Light Source, SLAC National Accelerator Laboratory, Menlo Park, CA 94025 USA; 7 Sorbonne Université, CNRS-INSU, Institut des Sciences de la Terre Paris, F-75005 Paris, France; 8 Atomic Weapons Establishment, Aldermaston, Reading RG7 4PR, United Kingdom; 9 Departments of Mechanical Engineering, Physics and Astronomy, and Laboratory for Laser Energetics, University of Rochester, Rochester, NY 14627 USA

## Abstract

Bismuth has long been a prototypical system for investigating phase transformations and melting at high pressure. Despite decades of experimental study, however, the lattice-level response of Bi to rapid (shock) compression and the relationship between structures occurring dynamically and those observed during slow (static) compression, are still not clearly understood. We have determined the structural response of shock-compressed Bi to 68 GPa using femtosecond X-ray diffraction, thereby revealing the phase transition sequence and equation-of-state in unprecedented detail for the first time. We show that shocked-Bi exhibits a marked departure from equilibrium behavior - the incommensurate Bi-III phase is not observed, but rather a new metastable phase, and the Bi-V phase is formed at significantly lower pressures compared to static compression studies. We also directly measure structural changes in a shocked liquid for the first time. These observations reveal new behaviour in the solid and liquid phases of a shocked material and give important insights into the validity of comparing static and dynamic datasets.

## Introduction

When compressed and heated, materials frequently undergo phase transitions associated with atomic structural changes to denser crystalline or amorphous (e.g. liquid) forms. Such high-pressure transformations, and the properties of the high-pressure phases, can be studied using X-ray diffraction on statically-compressed samples^1,2^, and, more recently, using rapid, or dynamic, compression coupled to powerful X-ray sources^3–6^. The rapid timescale of dynamic compression has led to questions about the kinetics of structural change - and whether dynamically-produced states are at thermodynamic equilibrium. Establishing the kinetics of phase transformations^3,4,6^, particularly those to the high-pressure phases encountered in dynamic compression^5–7^, is essential for a range of natural and technical applications from the study of meteor impact to the performance of alloys under high strain rates. Using an XFEL source, we have determined the lattice-level response of a shocked material as it undergoes multiple structural changes in both the solid and liquid phases, revealing a wealth of new behaviour.

Bismuth, together with iron, is an archetypal metallic phase-transforming material whose study has deepened our understanding on how materials respond to extreme compression^8^. Static compression studies at 300 K have revealed three structural transitions at 2.5, 2.8 GPa and 7.7 GPa^1^ with several complex structures forming, most notably the incommensurate structure of Bi-III (stable between 2.8–7.7 GPa in static compression studies). Despite Bi having also been studied extensively using shock compression^9–12^, its behavior under rapid pressure loading remains insufficiently understood. Phase transformations have been reported in shocked-Bi at 2.7 GPa^9^, 3.2 GPa^13^ and 7.0 GPa^11^, in reasonable agreement with the static solid-solid transition pressures if the raised temperatures attained in shock compression are accounted for. As traditional shock experiments provide no structural information, the identities of theses phases could only be assumed to be the same as the static phases, implying the transformations occur at, or close to, thermodynamic equilibrium. However, the densities of the high-pressure phases observed in the shock experiments are at considerable odds with those obtained in the static compression studies, with discrepancies as large as 5% at only ~5 GPa, and gradual density changes are seen^11^, suggestive of mixed-phase regions. As a result, it is not at all clear whether the complex structures observed in static compression studies on the timescales of seconds or minutes, such as incommensurate Bi-III, are the same as those that form in nanoseconds or microseconds under shock compression. Direct structural information on shocked-Bi has been limited, with low-fluence X-ray probes leading to only tentative phase assignments that largely adhere to the existing interpretation of equilibrium phase diagram^12^.

Similarly, while the melting behavior of Bi upon dynamic loading and unloading has been the subject of numerous theoretical and experimental studies^14–16^, nothing is known about the structure of the liquid at the very high-pressure conditions (>8 GPa) where melting is predicted to occur under shock compression. Given the number of solid-state phase transformations, and known liquid structural changes at lower pressure^17,18^, there is a pressing need to establish liquid structural properties at higher pressures such as above the shock melting point. While the presence of shocked liquid-Bi on release near 5 GPa was recently observed for the first time at an XFEL^4^, the limited angular range of the X-ray data precluded quantitative analysis of the liquid structure similar to that developed in static experiments^19^. However, the improved data quality and extended angular range that have since become available^5^ now allow for structural studies of shocked liquids for the first time.

## Results

**Figure 1:**
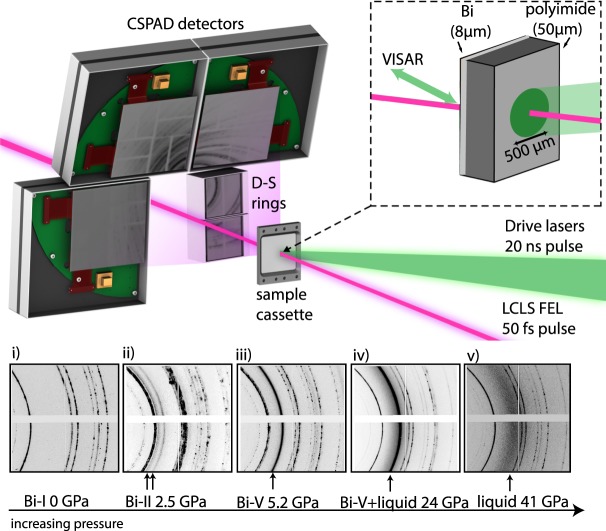
Experimental Setup and 2D diffraction data. (**a**) CSPAD detectors were arranged in a transmission Debye-Scherrer geometry in the MEC vacuum chamber. Dual drive beams were incident on target at angle of 15° and the XFEL beam probed the target at 30° from the target normal. The VISAR laser probed normal to the rear surface of the target. (**b**) 2D raw diffraction images from three different solid phases of Bi obtained on compression (profiles (i–iii)) and the liquid phase (profiles (iv,v)). The initial microstructure of the Bi starting material is retained as the Bi-I phase is compressed, but it drastically changed through the reconstructive phase transitions (profiles (ii,iii)). The strongest diffraction peaks from the high-pressure phase are indicated with arrows. Diffraction peaks from uncompressed Bi-I were observed in all diffraction profiles obtained before the shock reached the target rear surface.

All experiments were carried out at the Matter at Extreme Conditions (MEC) end station of the Linac Coherent Light Source (LCLS) XFEL. The experimental set up is shown in Fig. 1. Polycrystalline Bi samples of 8 *μ*m thickness were compressed using a Nd:glass drive laser, and the XFEL X-ray pulse was timed to probe the sample when the majority of it was in the peak compressed state. The VISAR (velocity interferometer system for any reflector) diagnostic measured the sample rear surface velocity history [see methods].

**Figure 2:**
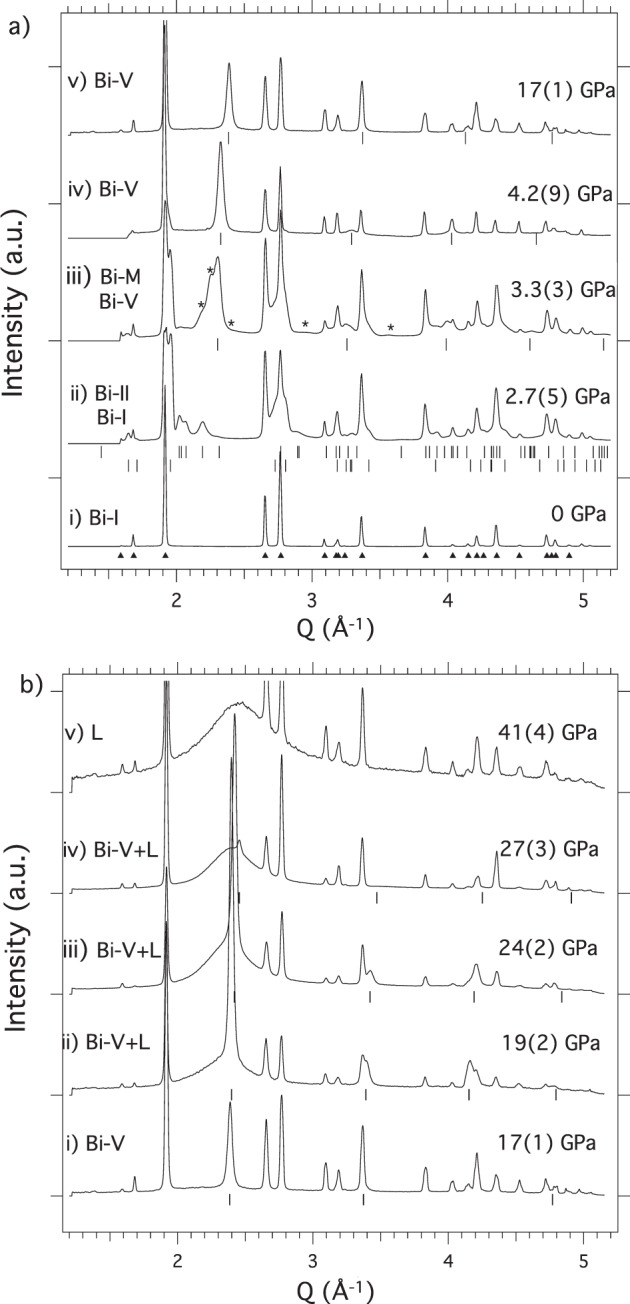
Solid phases + liquid phase (L) of Bi on shock compression. (**a**) Bi-I (profile (i)) transforms to Bi-II (profile (ii)) at 2.5 GPa (upper tick marks) with compressed Bi-I also being observed (lower tick marks). A transformation to a metastable phase (Bi-M, and whose peaks are marked with asterisks) is observed at 3 GPa (profile (iii)) and is always accompanied by diffraction peaks from bcc Bi-V, the locations of which are indicated by tick marks. Above 4 GPa only peaks from Bi-V are observed (profiles (iv,v)). (**b**) The first liquid scattering appears at 19 GPa (profile (ii)). The liquid phase-fraction grows with increasing pressure (profiles (iii,iv)) until only diffraction from the liquid phase is observed above 27 GPa (profile (v)). Peaks from uncompressed Bi-I, the locations of which are shown using triangles below profile (a)(i), are observed at all pressures.

Bi is observed to exist in 4 different solid phases on shock compression up to 17 GPa (Fig. 2a), above which the liquid phase emerges (Fig. 2b). Diffraction from the compressed Bi-I phase (space group $R\bar{3}m$, Z = 2) is always observed on shock compression at relatively low pressures (<9 GPa) as a result of the splitting of the shock front as Bi-I transforms to denser phases (Fig. 2a(ii)). The first solid-solid transformation is determined to occur at 2.5 GPa (Fig. 2a(ii)) and the Bragg reflections from the new phase fit very well to the structure of Bi-II (space group *C*2/*m*, Z = 4), with refined lattice parameters in excellent agreement with previous diamond anvil cell (DAC) studies of Bi-II at the same pressure^1^ (See Fig. S3).

However, a marked departure from equilibrium behavior is observed between 3–4 GPa as another phase transformation occurs (Fig. 2a(iii)), giving rise to a new set of Bragg reflections which cannot be fitted with the incommensurate Bi-III structure (stable between 2.8 and 7.7 GPa at 300 K in static compression studies). Several of the new Bragg reflections can be fitted to the body-centered cubic Bi-V phase (identified by tick marks in Fig. 2a(iii)) but there remain four additional reflections (highlighted with asterisks in Fig. 2a(iii)), which are observed reproducibly over a pressure region of 3–4 GPa which cannot be fitted to any equilibrium phase of Bi, including the high-temperature Bi-IV phase (see discussion in Supplementary Materials and Fig. S4). Thus, a metastable phase has formed. Definitive resolution of the structure of this new phase - termed Bi-M - is difficult due to the extensive peak overlap present in the 4-phase (ambient Bi-I, compressed Bi-I, Bi-M, Bi-V) profile of Fig. 2a(iii).

The Bragg reflections from the metastable phase disappear above 4 GPa, and thereafter the Bi-V phase is observed up to 17 GPa (Fig. 2b(i)). At 19 GPa, diffuse, textureless diffraction emerges together with diffraction from the Bi-V phase (Fig. 2b(ii)) - the Bi sample starts to melt. The liquid phase fraction grows as the pressure increases further (Fig. 2b(iii)), until, at 27 GPa (Fig. 2b(iv)), only a very small fraction of the Bi-V phase is observed. This pressure range over which solid-liquid coexistence occurs under shock compression, is in excellent agreement with the 18–27 GPa pressure range calculated by Pellisier *et al*.^14^.

**Figure 3:**
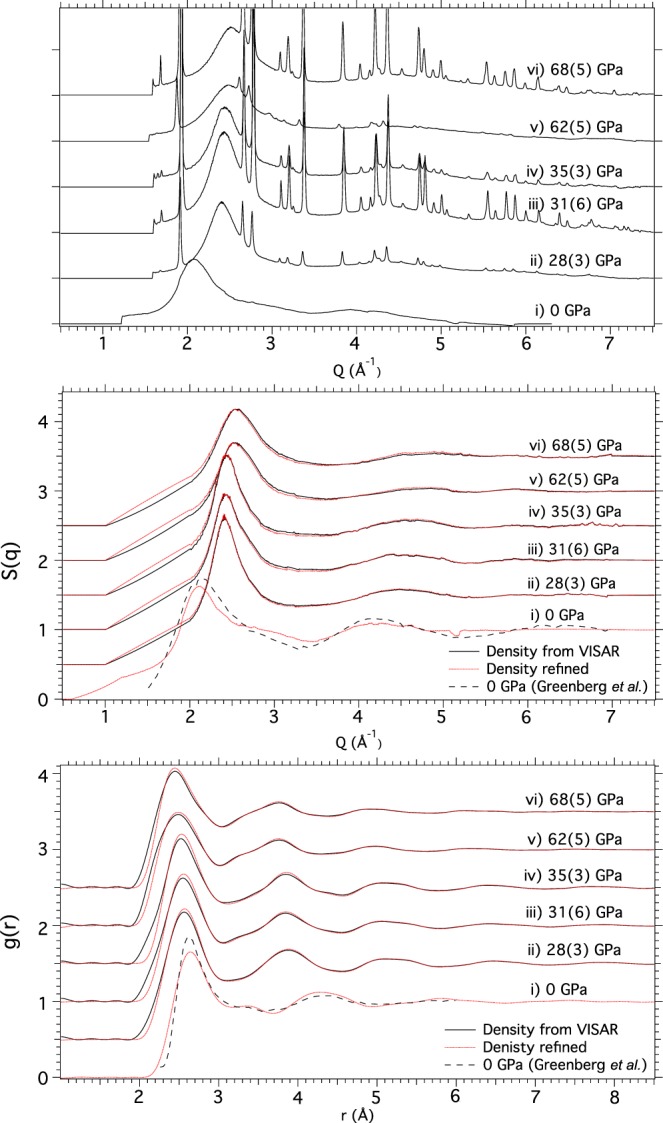
Local Structure of Liquid-Bi on Shock Compression (profiles spaced in intensity for clarity). (**a**) Integrated diffraction profiles from liquid-Bi from 28–68 GPa (profiles (ii–vi)), and also at 0 GPa and ~1500 K (profile (i)). (**b**) The corresponding *S*(*Q*) data. The prominent shoulder on the high-*Q* side of the first liquid peak at 0 GPa suggest that the local structure of liquid-Bi at this pressure deviates from that of a simple liquid. At higher pressures (profiles (ii–vi)) this shoulder is not observed, demonstrating that liquid-Bi is more simple and close-packed at these pressures. (**c**) The corresponding *g*(*r*) data. The form of *g*(*r*) changes as a shoulder on the left-hand side of the second coordination maximum becomes more prominent with increasing pressure, indicating liquid-Bi goes through a subtle and smooth transition with pressure. The full and dotted profiles in (**b**,**c**) show the *S*(*q*) and *g*(*r*) results obtained using sample densities determined using two different methods. The ambient pressure *S*(*q*) and *g*(*r*) from Greenberg *et al*. are shown by dashed line in (**b**)(i) and (**c**)(i) for comparison^23^.

At higher pressures, only liquid diffraction is observed (Fig. 3a). Three maxima in the liquid diffraction profiles are observed, all of which shift to higher-*Q* with pressure (compare Fig. 3a(i) with Fig. 3a(v)). The atomic structure factor *S*(*Q*) = 1 + 4$\pi {\rho }_{0}\,{\int }_{0}^{\infty }\,{r}^{2}[g(r)-1]\frac{\sin ({Q}_{r})}{{Q}_{r}}dr$, where *ρ*_0_ is the average atomic density, is obtained by scaling the diffracted intensity by the atomic scattering factor^20^ and normalising it to 1 at the largest experimental *Q* value (*Q* = $\tfrac{4\pi \,\sin (\theta )}{\lambda }$) (Fig. 3b(i–v)).

The ratio of the location of the first (*Q*_1_) and second (*Q*_2_) liquid maxima in the structure factor (*S*(*Q*)) can indicate the structural complexity of a liquid^21^. A close-packed liquid of hard spheres has symmetric peaks and a *Q*_2_/*Q*_1_ ~ 1.8 with this value increasing with structural asymmetry. Structural changes in the liquid state are most evident in the pair distribution function (*g*(*r*)), from which the coordination number ($CN=4\pi {\rho }_{0}{\int }_{0}^{{r}_{{\min }}}\,{r}^{2}g(r)dr$, where *r*_*min*_ is the location of the first minimum in *g*(*r*)) can be extracted. By adapting a Fourier analysis procedure developed by Eggert *et al*.^19^ we can also determine the density of liquid-Bi from the diffraction data alone. The accuracy of these values can be assessed by comparing with densities determined from the VISAR diagnostic. Pair distribution functions were generated by both allowing the density parameter to vary (red dotted lines in Fig. 3c) and fixing to the measured VISAR value (black lines in Fig. 3c). The agreement between the two functions in each case is very good.

By probing a Bi sample after it had completely released from peak pressure, we were also able to study the structure of liquid Bi at ambient pressure and ~1500 K. Our results show that the *Q*_2_/*Q*_1_ ratio of ~2 (Fig. 3b(i)), the form of the *g*(*r*) (Fig. 3c(i)), and the obtained CN of ~8 (Fig. S5) at these conditions all reproduce ambient-pressure data from previous static studies^22,23^. This value of coordination shows that liquid-Bi exists in an open, complex arrangement mirroring the complexity of the underlying solid phases at these conditions. The CN increases to ~12 by 28 GPa and the *Q*_2_/*Q*_1_ ratio decreases to ~1.8, where both remain constant thereafter up to 68 GPa (Fig. S5) demonstrating that liquid-Bi adopts a more close-packed structure at high-pressure, also matching results from static experiments^17,24^. However, inspection of the form of the *g*(*r*) between 35–63 GPa reveals an asymmetry in the second coordination shell emerging at ~4.5 Å indicating that the molten Bi has undergone a change in local structure (profiles (iv), (v), and (vi) of Fig. 3c). This feature in the *g*(*r*) is found not to be an artifact of the analysis procedure as it is observed whether the sample density is set as a refineable parameter in the *g*(*r*) determination, or whether the density is fixed at the value determined by VISAR. This structural change is observed through the shortening and splitting of the second coordination shell, from 5.70 Å in the low-pressure melts (*i*.*e*. 28–35 GPa) to 5.55 Å with a shoulder on the low-*r* side ~4.5 Å in the high-pressure melts (i.e. 62–68 GPa). Such a structural change transition is analogous to a transition from a close-packed/hard sphere liquid to a more complex structured liquid and could indicate an underlying, high-temperature phase transition in the solid phase. Our results in density are in agreement with the values determined by the VISAR diagnostic to better the 10% at the highest pressures. These uncertainties would be considerably reduced with the use of a higher-energy X-ray source (See Supp. Mat. for discussion of the liquid density analysis).

## Discussion

The shock compression behavior of Bi observed *via* X-ray diffraction differs from that reported from previous studies both in terms of the phases observed and conditions achieved. Rather than the smooth increase in density determined previously from changes in the observed shock wave profile, our ability to resolve distinct phases as a function of pressure reveals that Bi compresses through a sequence of first-order transitions closely resembling that seen in previous DAC data. The large discrepancies between our compression data and those of previous shock studies in the pressure range of 2.5–10 GPa are likely due to the difficulty in determining the sample densities accurately from shock wave-profile analyses due to the complications with dealing with multiple propagating waves in transforming materials^25^. By determining the structure of the high-pressure phases formed in Bi under shock compression, we can measure the density of these high-pressures phases in unprecedented detail overcoming the challenges faced by traditional shock experiments.

**Figure 4:**
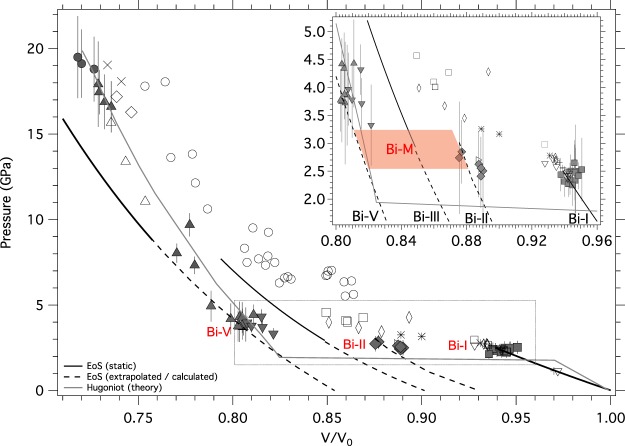
Multiphase Hugoniot of Bi. Compressibility data obtained in the current study are plotted using grey shaded symbols, and their phase is identified with red type. The peak sample pressure was determined using the *U*_*FS*_ as measured by VISAR and the volumetric compression was determined by diffraction (see pressure determination). The shaded lozenge in the inset indicates the region of *P* − *V* space to which our data constrain the metastable Bi-M phase. Data from Bi-V which were obtained from diffraction profiles containing diffraction peaks from both Bi-V and Bi-M are indicated with shaded inverted triangles. Previous shock compression data (including the data of Romain *et al*. (grey open circles)^11^) are shown by open symbols (triangles^15^, inverted triangles (^36^, squares (^8^, diamonds^9^, plus signs^37^, crosses^38^, and asterisks (reanalysed from^36^). The room temperature EoSs obtained from static-compression studies (labelled in the inset) are shown with solid black lines, and their extrapolation to ambient pressure with dashed lines. The EoS for Bi-II is taken from the computational study of Hausserman *et al*.^39^. The theoretical Hugoniot of Pelissier *et al*.^14^ (solid grey line) shows excellent agreement with our data throughout the entire pressure range studied. For tabular data see Table S1.

We observe Bi-V at conditions where it was previously thought to be elastically unstable under dynamic compression^16^. The measured lattice parameter of the Bi-V phase at these low pressures is typically *a* = 3.87 Å (*V*/*V*_0_ = 0.82), entirely consistent with that estimated by extrapolating the room-temperature static compression data for Bi-V down to this pressure^26^ (Fig. 4). The Bi-V phase has been observed in static compression experiments at 4 GPa and 550 K^22^ and so the observation of Bi-V in our experiments at unexpectedly low pressures might result from the sample being heated to elevated temperatures during shock compression. However, several studies which have predicted the pressure-temperature path of Bi under shock compression, and have shown that the heating of the sample during shock compression is negligible, largely due to a significant temperature decrease during the Bi-I-II transition^10,27^ associated with latent heat. Shock-induced heating to 550 K at 4 GPa, only 50 K below the melting temperature, would also result in melting at only slightly higher pressures, rather than at 19 GPa as observed here. Observing Bi-V at lower pressures is in stark contrast with standard models and observations for high-pressure phase transitions under dynamic loading, in which transition pressures are often elevated (over-driven) due to rate-limited ‘kinetic hindrance’^28,29^. The under pressurisation of Bi-V may instead be due to kinetic hindrance of the preceding phase (Bi-III), which causes the next-most stable phase to appear. In other words, the energy landscape of Bi under dynamic compression is, on the nanosecond timescale of the present experiment, distinct from the equilibrium landscape. Such an assertion is supported by a recent study on shock compressed Si which demonstrated how dynamic compression can access transformation pathways not observed in near equilibrium static compression studies^30^. However, Bi-III has been observed under dynamic compression in the dynamic DAC at compression rates of 102 GPa/s^31^, 7 orders of magnitude slower than the strain rates achieved in our experiments. A transition in the energy landscape may thus lie somewhere between nanosecond and millisecond timescales, a regime which might be accessible by gun-impact. Bi-M, then, is the next-most stable phase of Bi in a small pressure range, and/or a structural intermediary/precursor to Bi-III, on nanosecond timescales.

The absence of the incommensurate host-guest structure of Bi-III is unexpected, especially since a very similar structure has been observed in shock-compressed Sc^5^. The formation of composite, intermediate structures (as observed in the group-V metals) has been reported to result from structural frustration between competing structural processes in the Bi-I to bcc Bi-V transition path^32^. The combination of high pressurisation rates (10^9^ GPa/s) and significant shear forces associated with shock compression may be key in relieving this frustration, allowing the higher-symmetry Bi-V phase to form at only ~3 GPa. This result has implications for the many other phase transforming systems which form complex intermediate structures before transforming to a higher symmetry cubic phase such as As, Sb, Sn and Te.

In the liquid phase, group V elements have been shown to undergo structural changes, the most dramatic of which occurs in liquid-P which undergoes a first-order liquid-liquid phase transition at 1 GPa and 1300 K^33^. The structure of liquid-As has been shown to reflect the structural changes of the solid (A7 to simple cubic) with an increase in coordination number of 3 to 6 with pressure^34^. Our results (CN increasing from 8 to 12 with pressure) are analogous to the results of As, demonstrating that liquid-Bi too becomes more close-packed with pressure, consistent with the results of Yaoita *et al*.^24^. Observing subtle changes in liquid structure between 35–63 GPa demonstrates the high-quality data now achievable by coupling XFEL sources to high-power laser systems. Studying liquid structures using dynamic compression overcomes a major difficulty faced in static compression experiments as the large X-ray background generated by Compton scattering from the diamond anvils is eliminated, greatly reducing the experimental background level^19^.

By combining velocimetry measurements with high-quality diffraction from the LCLS, we have determined the multiphase behavior of Bi under shock compression to 68 GPa. Bi is observed to exhibit a marked departure from equilibrium behaviour, highlighting the inadequacy of relying on the static phase diagram to interpret dynamic compression results in phase transforming materials. The formation of the cubic Bi-V phase at 3 GPa in place of the complex Bi-III structure suggests that in nanosecond compression experiments the rich structural complexity exhibited by many phase-transforming elements may be significantly reduced or inhibited. We also resolved several gradual structural and density changes in the liquid phase of Bi with pressure which heralds the possibility of quantitatively studying liquid-liquid phase transitions relevant to planetary interiors for the first time.

## Methods

A Nd:glass optical laser (527 nm, 20 ns quasi-flat-topped pulse duration) was used to launch an ablation-driven shock wave through the samples. Both 250 *μ*m and 500 *μ*m hybrid phase plates were used to ensure the sample was irradiated by a smooth and consistent laser intensity profile. The densities of the 50 *μ*m polyimide ablator and 8 *μ*m Bi starting material were 1.42 g/cc and 9.8 g/cc respectively. To probe the behaviour of Bi below ~3 GPa the phase plates were removed and the beam was defocused to a diameter of 1 mm. In all cases, the compressed region was greater than the 260 *μ*m field of view of the VISAR and the quality of drive planarity was assessed from the planarity of the break out in the free surface/particle velocity history, as measured by the VISAR. Variations in break out time were generally less then 2% over the 50 × 50 *μ*m^2^ region probed by the X-ray beam (red shaded box in Fig. S1 top). The free surface velocity is then extracted using the fast Fourier transform method (FFT) (Fig. S1 bottom)^35^. Two VISAR legs where employed to resolve fringe-jump ambiguity from the compressed sample velocity traces. The LCLS provided quasi-monochromatic (ΔE/E = 0.5%) 8.8 or 10.0 keV X-ray pulses of 80 fs duration and with ~10^12^ photons per pulse. The X-ray beam was focused to 50 × 50 *μ*m^2^ and then centered on the focal-spot of the drive lasers, which, in turn, was centered on the target. Three CSPAD Quad 560k detectors and two smaller CSPAD 140k detectors were arranged in a transmission geometry to provide a two-theta range of ~20–110° and partial azimuthal coverage from ~−150° to 150°. The samples, which were cut into 2 mm squares were glued onto three 9 cm × 6 cm Al target holder plates before being mounted onto the MEC target holder and placed in the target chamber. Several X-ray calibrants, such as CeO_2_ and LaB_6_, were also loaded onto the target mount to determine the sample-to-detector distances and tilts. In addition, a fluorescent YAG (Yttrium Aluminium Garnet) sample was loaded in order to align the drive lasers with the VISAR laser. The VISAR collection duration was 50 ns.

## Electronic supplementary material

Supplementary Materials
