# Supplementary material for: Femtosecond diffraction studies of solid and liquid phase changes in shock-compressed bismuth
Source: Sci Rep. 2018 Nov 16;8:16927. doi: 10.1038/s41598-018-35260-3 (PMC6240068; doi:10.1038/s41598-018-35260-3)
Supplement: Supplementary file 1 — Supplementary Materials [file 41598_2018_35260_MOESM1_ESM.pdf]

Femtosecond diffraction studies of solid and liquid phase changes in shock compressed bismuth

M. G. Gorman,[1,2] A. L. Coleman,[1] R. Briggs,[1] R. S. McWilliams,[1] D. McGonegle,[3] C. A. Bolme,[4] A. E. Gleason,[4,5] E. Galtier,[6] H. J. Lee,[6] E. Granados,[6] M. Śilwa,[3] C. Sanloup,[7] S. Rothman,[8] D. Fratanduono,[2] R. F. Smith,[2] G. W. Collins,[9] J. H. Eggert,[2] J. S. Wark,[3] and M. I. McMahon[1]

[1]*SUPA, School of Physics & Astronomy, and Centre for Science at Extreme Conditions, The University of Edinburgh, Edinburgh, EH9 3FD, UK*
[2]*Lawrence Livermore National Laboratory, 7000 East Avenue, Livermore CA 94500, USA*
[3]*Department of Physics, Clarendon Laboratory, Parks Road, University of Oxford, Oxford, OX1 3PU, UK*
[4]*Shock and Detonation Physics, Los Alamos National Laboratory, P.O. Box 1663, Los Alamos, New Mexico 87545, USA*
[5]*Stanford Institute for Materials and Energy Sciences, SLAC National Accelerator Laboratory, Menlo Park, California 94025 USA*
[6]*Linac Coherent Light Source, SLAC National Accelerator Laboratory, Menlo Park, CA 94025*
[7]*Sorbonne Université, CNRS-INSU, Institut des Sciences de la Terre Paris, F-75005 Paris, France*
[8]*Atomic Weapons Establishment, Aldermaston, Reading, RG7 4PR, United Kingdom*
[9]*Departments of Mechanical Engineering, Physics and Astronomy, and Laboratory for Laser Energetics, University of Rochester, Rochester NY 14627*

Pressure Determination

The VISAR diagnostic recorded the free surface velocity (U_{FS}) of the shocked Bi sample which was converted to particle velocity (u_p) by using the acoustic approximation $U_{FS} = 2u_p$. In addition, we measured the density of the compressed sample from our diffraction measurements. We therefore can use the peak particle velocity, combined with the measured density of the bulk sample, to determine the peak sample pressure using $P = \rho_0 U_S u_p$, where $U_S = \frac{u_p}{1-\rho_0/\rho_1}$ and ρ_0 and ρ_1 are the densities of the initial and first shocked state respectively.

A phase transition in Bi to a denser phase was often identified in the free surface velocity profile with the splitting of the shock front into two distinct waves. After converting to particle velocity as before, we denote u_{p1} as the peak particle velocity of the first wave corresponding to the transition pressure P_1, and u_{p2} as the peak velocity of the slower-moving phase transformation wave which compresses the sample to peak pressure P_2. Along with these velocity measurements, we also record the densities of the region of the sample at the transformation point, and the region in the new phase at peak pressure, from our diffraction measurements - denoted as ρ_1 and ρ_2, respectively. In such cases, the peak sample pressure is determined using the two-jump Rankine-Hugoniot relation. $P_2 = P_1 + \rho_0(U_{S2} - u_{p1})(u_{p2} - u_{p1})$, where $U_{S2} = \frac{u_{p2} - u_{p1}\frac{\rho_1}{\rho_2}}{1-\frac{\rho_1}{\rho_2}}$.

Measured uncertainties in the pressure arise predominantly from uncertainties in the free surface velocity profile (Fig. S1). Variations in the measured U_{FS} over the $\sim$100 μm of the VISAR field of view (total field of view 260 μm) were typically $\sim$10%. The uncertainty in pressure was also due to the variation in measured sample density which is determined from Rietveld fitting of the diffraction data to a unit cell but was typically less than $\sim$1%. Uncertainties in pressure for shots which displayed one wave / two waves in the velocity history were determined by propagating these errors through the one-jump / two-jump Rankine-Hugoniot relations and were typically 12% / 20%.

Fig. S2 shows free surface velocity traces for several shots with increasing peak pressure. A two-wave feature is obvious in all traces below 9.7 GPa. The transformation point (Fig. S2 a) occurs at around 0.23 km/s (see profiles b, c and d) and is accompanied in the diffraction data by diffraction from the compressed Bi-I phase at $\sim$2.5 GPa which is consistent with the upper limit of the Bi-I stability region from previous DAC experiments [1]. The second wave is higher in velocity and corresponds to the region of the sample which has transformed to a higher-pressure phase such as Bi-II (profile b) or Bi-V (profile d), as confirmed by the observation of diffraction from such phases in addition to that from compressed Bi-I. However, at higher peak velocities, (profile e) this two-wave feature disappears as the phase transition has now become overdriven and only diffraction from the compressed Bi-V phase is observed. This is consistent with the study of Romain *et al.* who found the two-wave feature to disappear at this pressure [2]. In the current study, the occurrence of a second solid-solid phase transformation in Bi was resolved from our diffraction data (*i.e.* diffraction from three distinct, compressed phases was observed) but we were not able to resolve the resulting 3-wave structure in the wave profile unambiguously. The small elastic precursor for Bi, [3] is neglected in our calculations. In a small number of cases the two wave structure was unable to be resolved in the VISAR wave profile, even though both the compressed Bi-I and a higher-pressure phase where observed in the diffraction

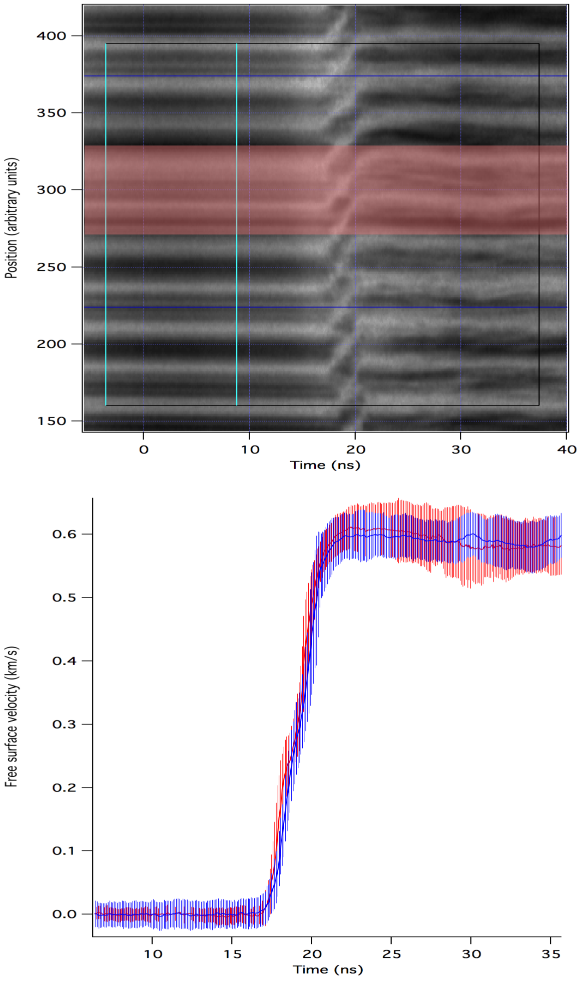

FIG. S1. Top: A typical VISAR image from one of the streak cameras. The red shaded area shows the region of the sample illuminated by the 50x50 μm^2 X-ray beam. Bottom: The corresponding free surface velocity trace which shows a two-wave feature. The peak velocity of the first and second wave are converted into u_{p1} and u_{p2} respectively by using the acoustic approximation $U_{FS} = 2u_p$. These two values are then combined with the measured density of the two phases ρ_1 and ρ_2 to give the pressure using the two-jump Rankine-Hugoniot relations. The standard deviations of velocity over the analyzed region are included.

profile. In this instance, for the purposes of pressure determination, the value of U_{FS} was assumed to be 0.23 km/s.

At pressures above 27 GPa the Bi sample melts completely to a non-reflecting liquid, and the free surface fringe motion cannot then be recorded. In such instances, we used an aluminised LiF window to contain the Bi sample, thereby allowing the fringe motion at the liquid-Bi/LiF interface to be recorded. Hugoniot parameters such as pressure and density in the Bi can then be determined by impedance matching with the LiF window using shock equations-of-state for LiF and Bi.

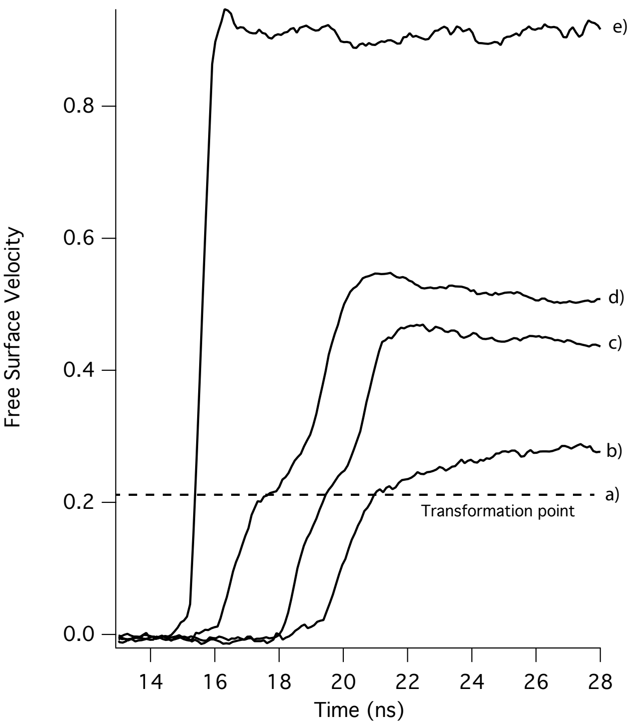

FIG. S2. Free surface velocity wave profiles obtained at (b) 2.7 GPa, (c) 3.3 GPa, (d) 4.4 GPa, (e) 9.7 GPa. A two-wave structure is observed at ~0.23 km/s up to at least 5 GPa, but is not observed above ~10 GPa (profile (e)) where the phase transition is overdriven. The wave profiles are shifted in time for clarity.

Microstructural analysis

The lack of peak asymmetry in the diffraction profiles from the crystalline Bi phases suggests each peak pressure state is well defined, and while the diffraction peaks from the high-pressure phases are broader than those from the uncompressed Bi, this arises from a reduction in the sample grain size through the reconstructive transition rather than any pressure-density gradients in the samples [4, 5]. While the oblique incidence of our x-ray probe to the shock propagation direction allows us to assess non-hydrostatic stresses (material strength) *via* azimuthally-anisotropic Debye-Scherrer rings [6], no detectable effect of strength [7] was observed in our diffraction patterns in the phase transition regime. We conclude that shear stresses have been relieved during the reconstructive phase transformation processes, and hence a direct comparison between our data may be made with those collected at hydrostatic equilibrium. The texture of the Bi starting material, as observed in the raw

diffraction images, proved useful in discriminating between those reflections which were from Bi-I and those which were from the higher-pressure phases. Diffraction from the ambient Bi-I phase is characterised by 'spotty' Debye-Scherrer rings (Fig 1.(i)) which were observed to retain their texture as the Bi-I phase was compressed (Fig. 1(ii)). This is contrary to our observations in other shock-compressed metals such as Sc [5], where the initial texture was lost immediately on compression. As the Bi transforms to higher-pressure phases (Fig. 1(iii) and (iv)) the new Debye-Scherer rings are both very smooth and broader, indicating that a significant microstructural rearrangement has occurred and a reduction in grain-size [4]. The strongest diffraction peaks from the high-pressure phase are indicated with arrows in Fig. 1b (ii-v). This loss of texture and peak broadening meant that Bragg reflections from Bi-I and the higher-pressure phases could be distinguished by visual inspection of the raw diffraction images alone.

The Bi-II phase

At 2.5 GPa, several new Bragg reflections are observed, which can be fitted to the monoclinic structure of Bi-II. Figure S3 is a three phase Rietveld fit to a diffraction profile at 2.5 GPa with the ambient Bi-I, compressed Bi-I and Bi-II structures. As can be seen, the fit is excellent with all observed reflections accounted for.

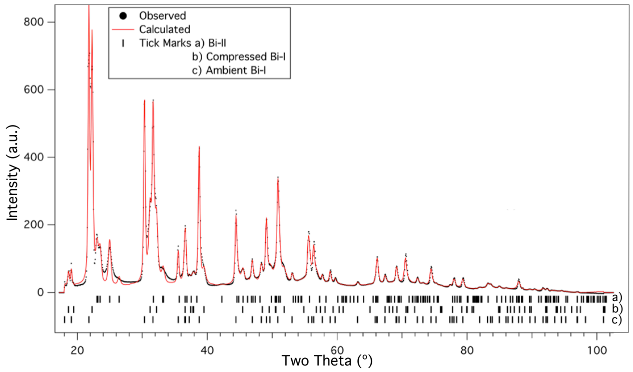

FIG. S3. Three phase Rietveld refinement to Bi diffraction data (black) of the Bi-II (a), compressed Bi-I (b) and ambient Bi-I (c) phases. The fit (red) is excellent. The X-ray probe wavelength was 1.24 Å

The metastable Bi-M phase

Peaks from the new metastable phase (Bi-M) are observed in all diffraction profiles collected over a narrow pressure range (3-4 GPa) and are always accompanied by peaks from Bi-V, suggesting that at these pressures Bi exhibits a subtle additional, (third) wave not clearly defined in our wave profiles (as observed in previous shock experiments [8]) or exists in a mixed phase. While the strongest of the peaks from Bi-M can be accounted for by the high-temperature Bi-IV phase, the medium-strength (422/512) reflection, which is predicted to be at $2\theta \sim 28°$, and which does not overlap with peaks from any of the other phases, is not observed (Fig. S4). In addition, the density obtained by fitting to the Bi-IV structure is greater than that of the Bi-V phase at the same pressure, and is in disagreement with measurements from DAC experiments at similar conditions by ∼8% [9]. We therefore conclude that the peaks do not come from Bi-IV or indeed any other previously observed phase of Bi, but that a previously-unobserved metastable phase has formed.

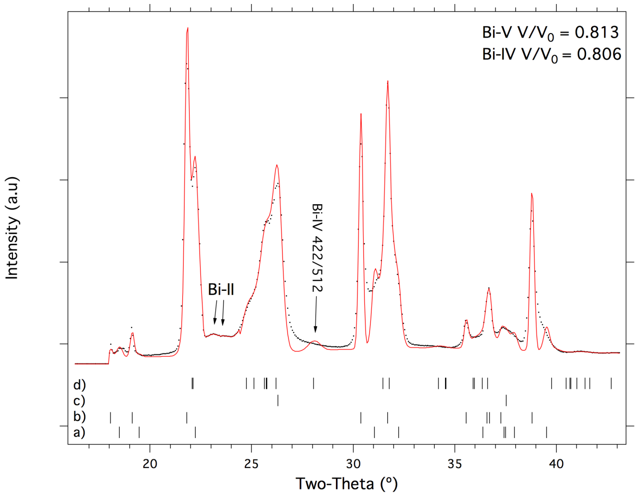

FIG. S4. Four-phase Rietveld refinement to Bi diffraction data (black) of the compressed Bi-I (a), ambient Bi-i (b), Bi-V (c) and Bi-IV (d) phases. While the fit (red) appears to the Bi-IV phase seems reasonable, the medium intensity (422/512) reflection is not observed. The determined lattice parameters are also not consistent with those from Bi-IV as the density is ∼ 8% greater than the same structure at similar conditions in the DAC. This fit also finds the Bi-IV phase to be 0.5% denser than the higher-pressure Bi-V phase. The X-ray probe wavelength was 1.24 Å

Liquid Analysis

The method to obtain the radial distribution function (RDF) and density of liquid Bi is described fully in Kaplow *et al.* [10] and Eggert *et al.* [11], whereby $g(r)$ is obtained by Fourier transforming the structure factor S(Q), *i.e.* the diffracted intensity scaled by the atomic scattering factor [12] and normalized to 1 at the largest experimental Q value (Q_{max}). The analysis procedure of Eggert *et al.* [11] developed for liquid diffraction measurements in diamond anvil cells to correct for

the large Compton scattering background from the diamond anvils is not considered here, as discussed in the main text. Bragg peaks from the uncompressed Bi-I phase were easily identified and removed from the liquid profiles before the analysis. Gaps between quadrants of the CSPADs, and the area close to the edges of the CSPADs which can give rise to 'hot' pixels, were masked out of the 1D integration of the diffraction data. Dark runs (CSPAD exposure was taken with the XFEL and drive laser off) were performed on every shot and subtracted from the measured data. Test exposures showed the background generated on the CSPADs by the 527 nm light from the drive lasers was negligible. A geometric correction to the measured intensity was applied to account for diffracted X-rays at different angles traveling through varying amounts of Al filtering on the detectors, the differing solid angle of each pixel, and the polarisation of the XFEL beam. We extracted the density of liquid Bi from the radial distribution function by performing the optimisation procedure discussed in Eggert *et al.* [11], where density is a refineable parameter which is varied iteratively until the normalised χ^2 value is minimised. The density of the bulk liquid sample was also determined on each shot from the VISAR measurements at the rear surface, and so the RDF was also generated by fixing the density parameter at the value measured by VISAR. Fig. 3c overlays the $g(r)$ determined for each method and, as can be seen, the form of the profiles are very similar. However, the density of the liquid Bi as determined from the optimisation procedure was found to be systematically less than that determined using the VISAR (6% less at 28 GPa and 10% less at 68 GPa). For example, the density obtained from VISAR at 28 GPa was 13.83 g/cc compared with 12.95 g/cc as determined from Fourier analysis. This disagreement in the measured density results from slight variations in the peak intensities in the $g(r)$ and are probably due to the limited angular range currently possible in our experiments. We do not collect diffraction data at low angles, meaning the $S(q)$ has to be extrapolated down to $S(0)$. The refined density also can vary by up to 10 % depending on the choice of extrapolation (e.g. linear, stepwise, polynomial) and so positioning detectors to measure data in this range would remedy this problem. Previous diamond anvil cell studies have determined an average density by determining the density from different Q cut off values. These values generally varied from 6.5 - 9 $\AA^{-1}$ with typical variations of around 10%. Our data only extends to 7.5 Å and thus such an averaging cannot be performed. This will be overcome in future studies by using higher energy X-ray energies.

Structure of Liquid Bi

At ambient pressure the Q_2/Q_1 ratio is observed to be ∼1.96 (filled squares in Fig. S5 (i) consistent with previous DAC (open squares in Fig. S5 (i)) [13] and large volume press studies (open circles in Fig. S4 (i)) [14]. This value decreases with pressure to ∼1.8 at 28 GPa where it remains approximately constant up to 68 GPa. The results suggest that liquid Bi exists in a more close-packed arrangement at high-pressure relative to ambient. The coordination number of liquid Bi is observed to change from ∼8 at ambient pressure to ∼12 at 28 GPa where it remains approximately constant up to 68 GPa Fig. S5 (ii). This trend of liquid Bi becoming more close packed with pressure is consistent with data from a previous DAC study [13] (open squares in Fig. S5 ii)).

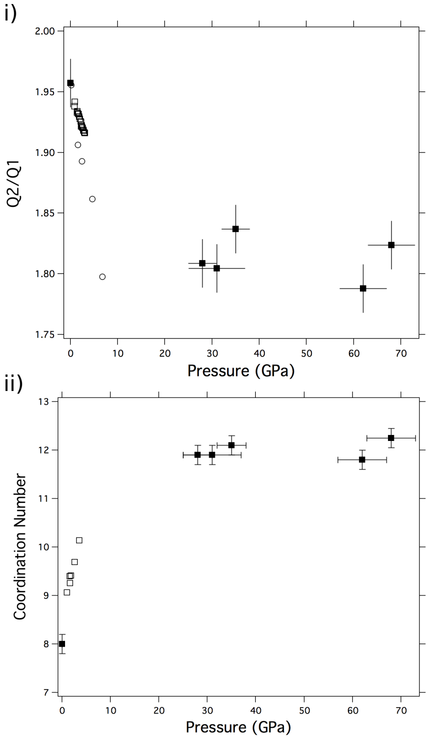

FIG. S5. Pressure dependence of the structure of liquid Bi under shock compression. i) Q_2/Q_1 as a function of pressure. Filled squares - our data, unfilled squares - previous DAC data [13], unfilled circles - previous large volume press data [14]. ii) Coordination number of liquid Bi as a function of pressure. Filled squares - our data, unfilled squares - previous DAC data [13].

[1] O. Degtyareva, M. I. McMahon, and R. J. Nelmes, High Pressure Research **24**, 319 (2007).
[2] J. P. Romain, Journal of Applied Physics **45**, 135 (1974).

TABLE S1. Table of P-V Hugoniot Data (Asterisks indicates shots where Bi-M phase is observed as well as diffraction from the Bi-V phase)

Phase	$\frac{V}{V_0}$	Pressure (GPa)
Bi-I	0.951(2)	2.5(2)
Bi-I	0.942(1)	2.3(2)
Bi-I	0.946(1)	2.5(2)
Bi-I	0.945(2)	2.2(2)
Bi-I	0.946(1)	2.3(2)
Bi-I	0.943(1)	2.4(2)
Bi-I	0.940(1)	2.3(2)
Bi-I	0.938(1)	2.4(2)
Bi-I	0.936(2)	2.1(2)
Bi-I	0.944(1)	2.5(2)
Bi-I	0.947(1)	2.5(2)
Bi-II	0.890(3)	2.5(4)
Bi-II	0.889(3)	2.4(2)
Bi-II	0.888(3)	2.6(3)
Bi-II	0.889(3)	2.6(4)
Bi-II	0.877(3)	2.8(4)
Bi-II	0.875(3)	2.7(5)
Bi-V*	0.822(1)	3.3(3)
Bi-V*	0.808(1)	4.0(4)
Bi-V*	0.810(1)	3.8(4)
Bi-V*	0.815(1)	4.3(4)
Bi-V*	0.816(1)	3.7(4)
Bi-V	0.807(1)	3.9(10)
Bi-V	0.811(1)	4.4(5)
Bi-V	0.806(1)	3.7(5)
Bi-V	0.805(1)	3.9(8)
Bi-V	0.788(1)	4.9(9)
Bi-V	0.804(1)	4.3(9)
Bi-V	0.804(1)	3.7(9)
Bi-V	0.805(1)	4.3(9)
Bi-V	0.799(1)	4.2(9)
Bi-V	0.803(1)	3.8(8)
Bi-V	0.770(1)	8.0(6)
Bi-V	0.777(1)	9.7(6)
Bi-V	0.780(1)	7.3(5)
Bi-V	0.736(1)	16.6(14)
Bi-V	0.732(1)	16.9(16)
Bi-V	0.729(1)	17.5(13)
Bi-V	0.729(1)	17.9(25)
Bi-V	0.720(1)	19.1(12)
Bi-V	0.718(1)	19.5(19)
Bi-V	0.727(1)	18.8(13)

[3] R. F. Smith, J. H. Eggert, M. D. Saculla, A. F. Jankowski, M. Bastea, D. G. Hicks, and G. W. Collins, Physical Review Letters **101**, 065701 (2008).

[4] A. E. Gleason, C. A. Bolme, H. J. Lee, B. Nagler, E. Galtier, D. Milathianaki, J. Hawreliak, R. G. Kraus, J. H. Eggert, D. E. Fratanduono, G. W. Collins, R. Sandberg, W. Yang, and W. L. Mao, Nature Communications **6**, ncomms9191 (2015).

[5] R. Briggs, M. G. Gorman, A. L. Coleman, R. S. McWilliams, E. E. McBride, D. McGonegle, J. S. Wark, L. Peacock, S. Rothman, S. G. Macleod, C. A. Bolme, A. E. Gleason, G. W. Collins, J. H. Eggert, D. E. Fratanduono, R. F. Smith, E. Galtier, E. Granados, H. J. Lee, B. Nagler, I. Nam, Z. Xing, and M. I. McMahon, Physical Review Letters **118**, 025501 (2017).

[6] A. Higginbotham and D. McGonegle, Journal of Applied Physics **115**, 174906 (2014).

[7] J. R. Asay, Journal of Applied Physics **48**, 2832 (1977).

[8] Z. Rosenberg, Journal of Applied Physics **56**, 3328 (1984).

[9] W. Chaimayo, L. F. Lundegaard, I. Loa, G. W. Stinton, A. R. Lennie, and M. I. McMahon, High Pressure Research **32**, 442 (2012).

[10] R. Kaplow, S. L. Strong, and B. L. Averbach, Physical Review **138**, A1336 (1965).

[11] J. H. Eggert, G. Weck, P. Loubeyre, and M. Mezouar, Physical Review B **65**, 174105 (2002).

[12] J. H. Hubbell, W. J. Veigele, E. A. Briggs, R. T. Brown, D. T. Cromer, and R. J. Howerton, Journal of Physical and Chemical Reference Data **4**, 471 (1975).

[13] C. Lin, J. S. Smith, S. V. Sinogeikin, Y. Kono, C. Park, C. Kenney-Benson, and G. Shen, Nature Communications **8**, 14260 (2017).

[14] K. Yaoita, K. Tsuji, Y. Katayama, M. Imai, J. Chen, T. Kikegawa, and O. Shimomura, Journal of Non-Crystalline Solids **150**, 25 (1992).
